# Origin of colossal dielectric permittivity of rutile Ti_0.9_In_0.05_Nb_0.05_O_2_: single crystal and polycrystalline

**DOI:** 10.1038/srep21478

**Published:** 2016-02-12

**Authors:** Yongli Song, Xianjie Wang, Yu Sui, Ziyi Liu, Yu Zhang, Hongsheng Zhan, Bingqian Song, Zhiguo Liu, Zhe Lv, Lei Tao, Jinke Tang

**Affiliations:** 1 Department of Physics, Harbin Institute of Technology, Harbin 150001, People’s Republic of China; 2 Department of Physics & Astronomy, University of Wyoming, Laramie, WY 82071, USA

## Abstract

In this paper, we investigated the dielectric properties of (In + Nb) co-doped rutile TiO_2_ single crystal and polycrystalline ceramics. Both of them showed colossal, up to 10^4^, dielectric permittivity at room temperature. The single crystal sample showed one dielectric relaxation process with a large dielectric loss. The voltage-dependence of dielectric permittivity and the impedance spectrum suggest that the high dielectric permittivity of single crystal originated from the surface barrier layer capacitor (SBLC). The impedance spectroscopy at different temperature confirmed that the (In + Nb) co-doped rutile TiO_2_ polycrystalline ceramic had semiconductor grains and insulating grain boundaries, and that the activation energies were calculated to be 0.052 eV and 0.35 eV for grain and grain boundary, respectively. The dielectric behavior and impedance spectrum of the polycrystalline ceramic sample indicated that the internal barrier layer capacitor (IBLC) mode made a major contribution to the high ceramic dielectric permittivity, instead of the electron-pinned defect-dipoles.

In recent years, the search for materials with colossal permittivity continues to attract considerable interest motivated by academic research and potential applications for smaller and faster electronics as well as high-energy-density storage^1^. There are many excellent colossal dielectric permittivity materials, such as doped BaTiO_3_^2^, CaCu_3_Ti_4_O_12_ (CCTO)^3^,^4^, doped NiO^5^, Bi_0.5_Na_0.5_TiO_3_^6^,Ni_0.5_Zn_0.5_Fe_2_O_4_^7^, ZnO^8^ etc., but the high permittivity of the ferroelectric materials, such as BaTiO_3_ and (K, Na)NbO_3,_ can only be achieved over a narrow temperature range close to the ferroelectric phase transition^9^,^10^. The stronger temperature dependence limits their applications. The dielectric loss of other non-ferroelectric materials with colossal values of ε is too high to apply. To make matters worse, it is very hard to achieve a balance between high ε and affordable low dielectric loss^11^.Recently, significant dielectric behavior was reported in (In + Nb) co-doped TiO_2_ polycrystalline ceramic^12^. The coexistence of colossal dielectric constant (CP) and low dielectric loss makes it attract considerable attention. Similar dielectric behavior was found in niobium and some other trivalent cations co-doped TiO_2_ polycrystalline ceramics^13^,^14^. The excellent dielectric properties in (In + Nb) co-doped TiO_2_amorphous thin films make wide application possible^15^.

A new mechanism named electron-pinned defect-dipoles was proposed by Hu *et al.* in order to explain the excellent dielectric properties^12^. They suggested that the local 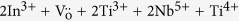 defect clusters were responsible for the colossal permittivity and low dielectric loss^12^. The colossal dielectric permittivity of amorphous film^15^ and nano-crystalline^16^ of (In + Nb) co-doped TiO_2_ samples support this theory, which is considered to be an essential property independent of microstructure. Liu *et al.* considered that electron-pinned defect-dipoles, interfacial polarization and polaron hopping polarization contribute to the colossal permittivity together^17^. Li *et al.* suggested that the semiconducting grains and insulating grain boundaries contribute to the colossal permittivity behavior of (In + Nb) co-doped rutile TiO_2_ ceramics samples, which is named the internal barrier layer capacitor (IBLC) effect. But they didn’t eliminate the role of electron-pinned defect-dipoles^18^,^19^,^20^.The origin of novel dielectric behavior for co-doped TiO_2_ ceramics is still controversial because the two proposed mechanisms result in very similar dielectric behaviors in polycrystalline ceramic. Therefore, the dielectric behaviors of (In + Nb) co-doped TiO_2_single crystal are very important data to clarify the issue.

In this paper, we firstly observed the colossal dielectric permittivity up to 10^4^ at room temperature of (In + Nb) co-doped rutile TiO_2_ single crystal and polycrystalline ceramic. The voltage-dependence of the dielectric permittivity and the impedance spectrum suggest that the high dielectric permittivity of single crystal originated from the surface barrier layer capacitor (SBLC). The temperature dependence of dielectric behavior and impedance spectrum of the polycrystalline ceramic sample indicate that the internal barrier layer capacitor (IBLC) mode made a major contribution to the high dielectric permittivity instead of the electron-pinned defect-dipoles.

## Results and discussions

**Figure 1:**
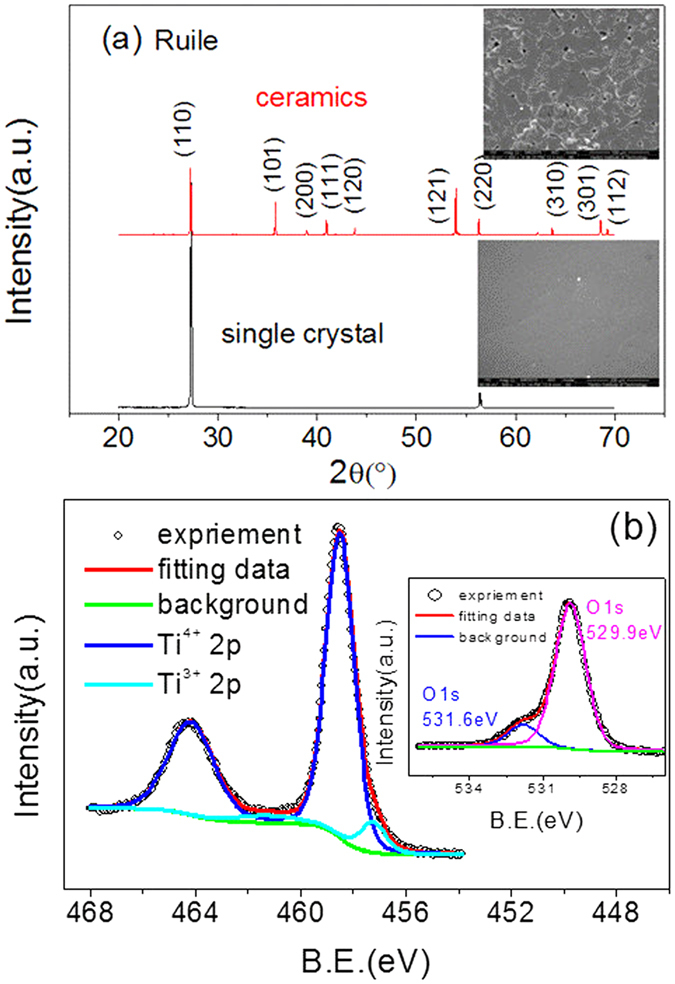
(**a**) XRD pattrens and SEM images of In_0.05_Nb_0.05_Ti_0.9_O_2_ single crystal (black line) and polycrystalline ceramics (red line); (**b**) Valence states of the elements Ti in the TiO_2_ single crystal co-doped with 10% (In + Nb), the insert shows the XPS data of O1s.

Fig. 1(a) shows the XRD of (In + Nb) co-doped TiO_2_ polycrystalline and single crystal. Both samples are in the pure rutile phase. The θ-2θ XRD scan of single crystal on the habit plane of the crystal exhibit two peaks of (110) and (220) in the 2θ region of 20–70°, meaning that a high quality single crystal was obtained. The FE-SEM images of the single and polycrystalline crystals were shown in the inset of Fig. 1(a). For the single crystal sample, a very smooth surface was observed in the 481×481 μm area indicating the absence of grain boundaries that may play a crucial role in many colossal dielectric permittivity materials^4^,^5^. A duplex microstructure consisting grains and grain boundaries was observed in the polycrystalline sample, and the size of the grains was about 20 μm. Thus, we have confirmed that there are a great number of grains and grain boundaries in polycrystalline ceramics, but not in single crystal. The mole ratio of In, Nb and Ti in both single crystal and ceramics are 1:1:18 with the consistent design as shown in Table S1.

Fig. 1(b) shows the XPS spectra for the surface of the 10% (In + Nb) co-doped TiO_2_ single crystal to identify the corresponding chemical valence states. Similar results had also been observed in our polycrystalline ceramics (shown in Fig. S1). The XPS results of Ti 2p in the single crystal reveals the existence of Ti^3+^ ions, giving a Ti^3+^/Ti proportion of ~5.7%, which is very similar to the ceramic results reported previously.^12^It is well-known that oxygen vacancies and Ti^3+^ can be produced by the doping of both niobium and trivalent cations in TiO_2_^21^,^22^. The inset of Fig. 1(b) shows the O1s XPS data of single crystal. The 529.9 eV peak position corresponds to the Ti-O bond in rutile TiO_2_ and the 531.1 eV peak position may be associated with the oxygen vacancy and surface hydroxyl groups^23^,^24^. The XPS result (not shown here) of In and Nb indicates that the chemical valence states are +3 and +5.

The process of the Ti^4+^ to Ti^3+^ reduction and the appearance of the oxygen vacancies in (In + Nb) co-doped TiO_2_ can be attributed to three processes in the following equations:


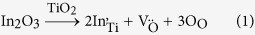



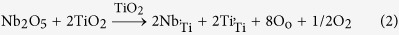



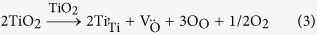


The In/Nb co-doping induces oxygen vacancies and Ti^3+^. Additionally, the oxygen vacancy caused from oxygen loss in the single crystal during growth can produce additional Ti^3+^, as given in equation (3). The necessary conditions to form 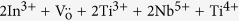 defect clusters is fully equipped in the co-doped rutile TiO_2_ single crystal, so the electron-pinned defect-dipoles theory may be demonstrated the dielectric behavior in the single crystal sample^12^,^15^.

**Figure 2:**
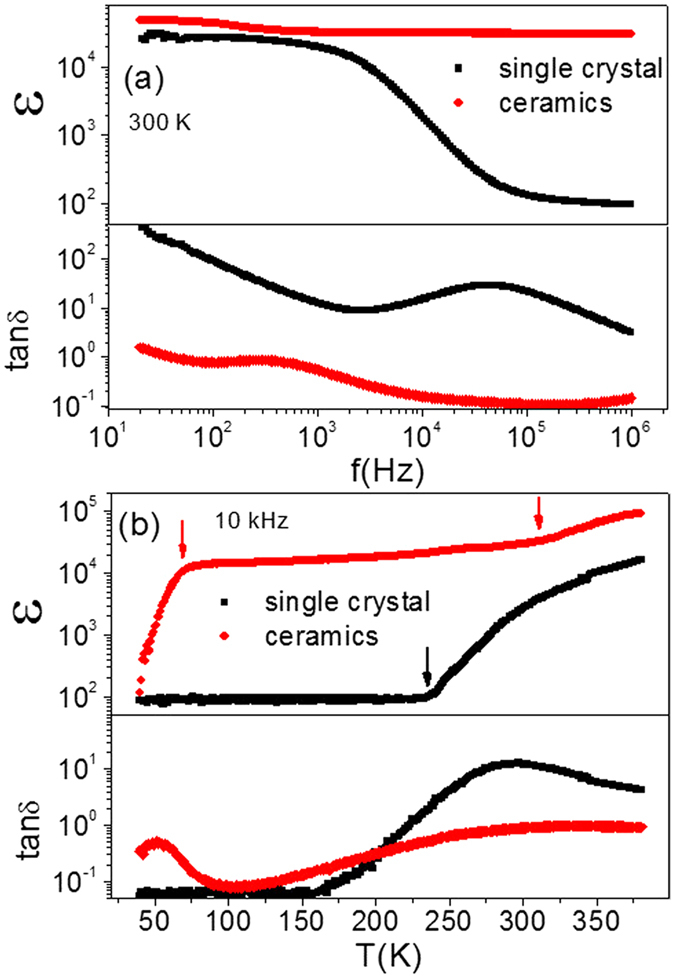
Dielectric constant and dielectric loss of 10% (In + Nb) co-doped rutile TiO_2_ single crystal and polycrystalline ceramics as a function of frequency (**a**) and temperature (**b**).

Fig. 2(a) shows the frequency dependence of the dielectric constant and dielectric loss of 10% (In + Nb) co-doped TiO_2_ single crystal and polycrystalline ceramics at room temperature. Fig. 2(b) shows the temperature dependent dielectric constant and dielectric loss. Interestingly, a colossal dielectric permittivity up to 10^4^ over the low frequency range was achieved for the (In + Nb) co-doped TiO_2_ single crystal, and dropped dramatically to ε~85 that was very close to the value of pure rutile TiO_2_ single crystal. Additionally, a dissipation peak appears between 10^4^ ~ 10^5^ Hz, which indicates a dielectric relaxation process. The dielectric permittivity quickly falls two orders magnitude from 10^4^ to 10^2^ with temperature decreased, and the dissipation peak of dielectric relaxation is observed at a temperature of 290 K. This result further confirms that one dielectric relaxation process in the (In + Nb) co-doped TiO_2_ single crystal.

For the ceramics sample, as shown in Fig. 2(a), a high dielectric permittivity, up to 45000, is observed in the low frequency range, and dropped to a lower dielectric permittivity, 30000, as the frequency increased up to 10^6^ Hz. A dissipation peak toward dielectric relaxation appears between 10^2^ ~ 10^3^ Hz. Consequently, there should be another dielectric relaxation process at a higher frequency range (>10^6^ Hz) at room temperature because the high dielectric permittivity, 30000, is much larger than that in pure rutile TiO_2_. The temperature dependent dielectric constant and dielectric loss tests were carried out to confirm this hypothesis, as shown in Fig. 2(b). The dielectric constant changes smoothly with decreasing of temperature and suddenly falls two orders of magnitude at 50 K. Additionally, two dissipation peaks are observed at 50 K and 320 K. Therefore, two dielectric relaxations exist in the polycrystalline ceramic while only one dielectric relaxation process exists in the single crystal.

Due to the absence of grain boundaries in single crystal, the high dielectric permittivity of (In + Nb) co-doped TiO_2_ single crystal in the low frequency range cannot be attributed to an internal barrier layer capacitor (IBLC). Therefore, the electron-pinned defect-dipoles theory proposed by Hu *et al.*^12^ and surface barrier layer capacitor (SBLC) mode that is confirmed to exist in a CaCu_3_Ti_4_O_12_(CCTO) single crystal^25^,^26^,^27^ are the alternative approach to get the high dielectric permittivity in (In + Nb) co-doped TiO_2_ single crystal. According to Hu’s theory, electrons hop in defect clusters and give high electronic conductivity, which can only be driven by thermal activation^12^,^14^. Consequently, the dielectric behavior should change little under different testing voltage. However, the dielectric permittivity decreased with the increasing testing voltage at low frequency range, which suggested that the CP of (In + Nb) co-doped TiO_2_ single crystal is caused by the SBLC because of the decreased of R_e_ (the resistance of electrode contact impedance between single crystal and electrode) under high voltage^25^. The relationship of dielectric permittivity and R_e_ can be described by equation (4, 5, 6, 7)^28^.


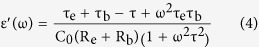



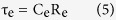



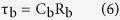



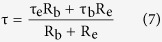


Here, C_0_ is a constant determined by the single crystal area and thickness. R_b_ is the bulk resistance. C_e_ and C_b_ are capacitance due to SBLC and bulk, where 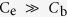
25. Considering this factor, equation (4) can be developed to equation (8).


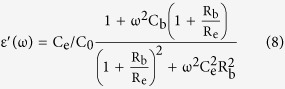


According to above equation, 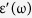 reduces with increased R_b_/R_e_, because 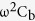 (C_b_~10^−12^ F) and 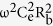 are much less than 1 over the frequency range where the high dielectric permittivity appears.

**Figure 3:**
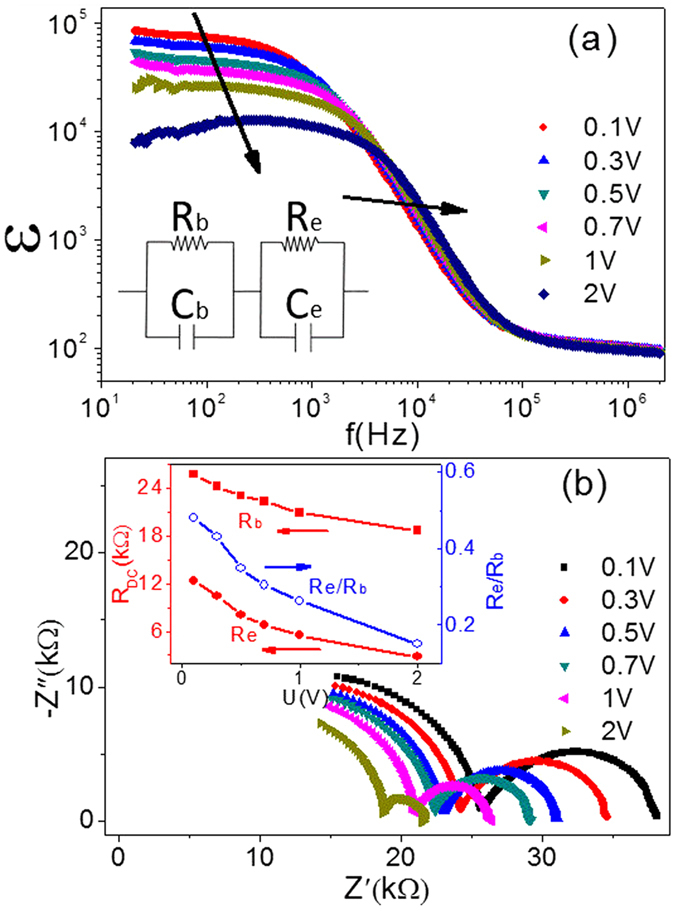
Impedance analysis and dielectric behavior of 10% (In + Nb) co-doped rutile TiO_2_ single crystal. (**a**) Dielectric constant as a function of frequency at different testing voltages, the equivalent electric circuit is shouwn in the insert. (**b**) Impedance spectroscopy at a different testing voltage, the R_b_, R_e_ and R_b_/R_e_under different testing voltage are shouwn in the insert.

Fig. 3(a) shows that the dielectric permittivity of single crystal sample in low frequency range declined and the relaxation time 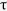 reduced continuously with the increased testing voltage. Here the equivalent circuit can be expressed in the inset of Fig. 3(a), in which, 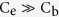
25. This point is supported by the impedance spectrum data shown in Fig. 3(b). For example, when the testing voltage is 1 V, C_e_ and C_b_ are about ~10^−8^ F and ~10^−12^ F, while R_e_ and R_b_ are 5.5 kΩ and 20.9 kΩ, respectively. As mentioned above, 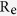 reduced rapidly with the increased of testing voltage. Although R_b_ also decreased at the same time, R_b_/R_e_ increased continuously in this process, as shown in the inset of Fig. 3(b). This is consistent with the SBLC model. In addition, the reduced of the bulk resistance may be ascribed to the injected electrons, which are trapped by oxygen vacancies^29^. This phenomenon again proves the existence of oxygen vacancies in single crystal sample. Therefore, the high dielectric permittivity of (In + Nb) co-doped TiO_2_ single crystal is attributed to the SBLC, and has no relationship with electron-pinned defect-dipoles.

**Figure 4:**
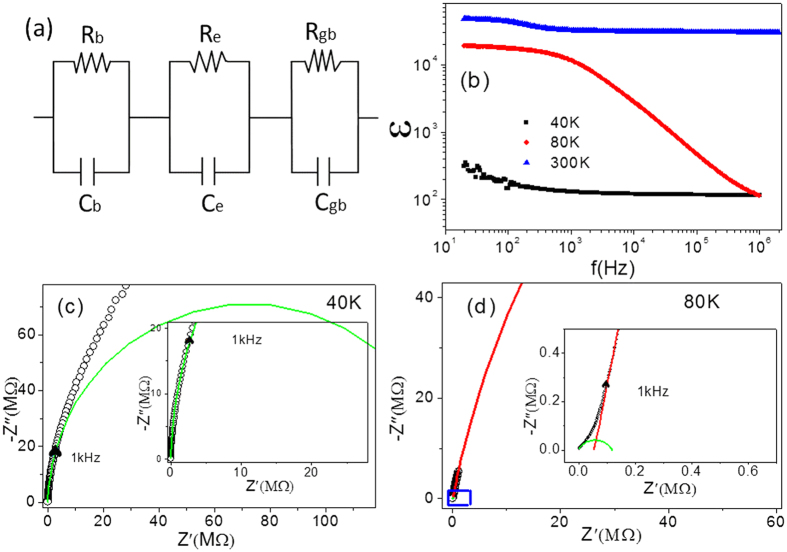
Impedance analysis and dielectric behavior of 10% (In + Nb) co-doped rutile TiO_2_ polycrystalline ceramics. (**a**) The equivalent electric circuit. (**b**) Dielectric constant as a function of frequency. (**c,d**) are Impedance spectroscopy at 40 K and 80 K respectively.The solid symbols are the experimental results. The solid lines are the best fitting results.

The data of 10% (In + Nb) co-doped TiO_2_ polycrystalline ceramic can be modeled on an equivalent circuit consisting of three parallel RC elements in series, as shown in Fig. 4(a).The first plateau of the dielectric permittivity is attributed to boundary (IBLC), owing to the appearance of the microstructure consisting of grain boundaries and grains compared to single crystal. Referring to the single crystal research results, the second dielectric permittivity plateau in the polycrystalline ceramic at low frequency can be attributed to the SBLC model. The dielectric permittivity should drop to the level of 10^2^ if the frequency was high enough (>10^6^ Hz), and then only the dielectric behavior of bulk contributes to the permittivity^25^. This phenomenon can’t be observed in the range of 10-10^6^ Hz at room temperature but at lower temperature, as shown in Fig. 4(b).

Fig. 4(b) and (c) show the frequency dependence of dielectric permittivity and the impedance spectroscopy at different temperature, respectively. According to this mode, there should be three arcs, of which the position in the frequency spectrum depends on their relaxation times τ, where τ = RC: the relationship 2πfτ = 1 works at the arc maxima, where f is the applied frequency. Generally speaking, 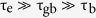
25, where 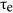, 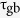 and 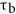 are relaxation time of electrode contact impedance, grain boundary impedance and bulk impedance. The dielectric relaxation time becomes smaller with the temperature increasing due to its’ decreased resistance. It often occurs that the three dielectric permittivity steps can’t be simultaneously achieved at a fixed temperature because the relaxation time is too large or too small compared to the applied frequency range. Meanwhile the impedance arcs show a similar behavior.

**Figure 5:**
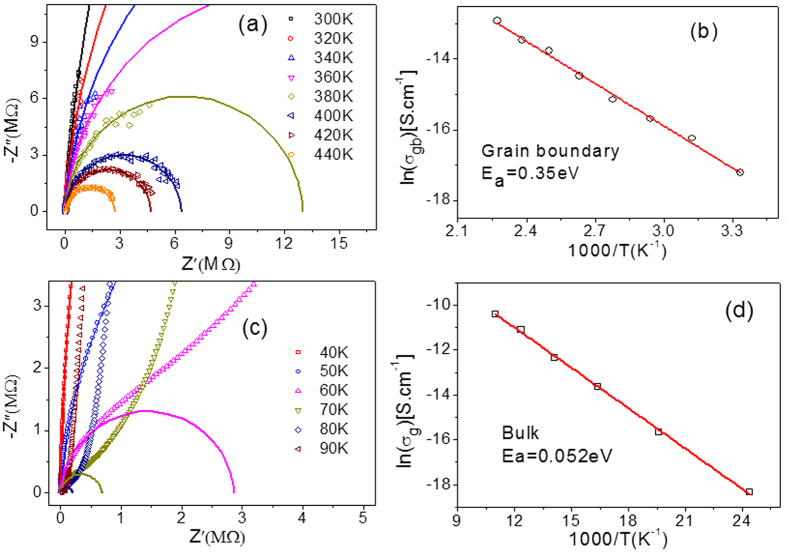
Impedance complex plane plots at different temperatures and arrhenius plots of bulk and grain boundary conductivity data for the polycrystalline ceramics. (**a**) Complex impedance plots of the polycrystalline ceramics from 300 to 440 K, which represents the grain boundary arc. (**b**) Temperature dependence of conductivity for the grain boundary. (**c**) Complex impedance plots of the polycrystalline ceramics from 40 to 90 K, which represents the grain arc when the frequency is high enough (fitting curve). (**d**) Temperature dependence of conductivity for the grain. The activation energies of the grain boundary and bulk are 0.35 eV and 0.052 eV, respectively.

The dielectric permittivity in the frequency range from 10^3^ to 10^6^ Hz is about 130 at 40 K. Therefore, the grain boundary response does not occur because τ_gb_ is much larger than 1/2πf (1.6*10^−7^~1.6*10^−4^ s).Correspondingly, only one arc, which is considered to be the bulk response, appears in impedance spectroscopy at 40 K during 10^3^ to 10^6^ Hz (as shown in Fig. 4(c)). The dielectric permittivity starts to ascend when the applied frequency is below 10^3^ Hz, and another arc thought to be the grain boundary response appears in the impedance spectrum. The dielectric permittivity reduced sharply from 10^4^ to 10^2^ magnitude in the frequency range from 20 to 10^6^ Hz with increasing frequency when the temperature was at 80 K; while the grain boundary response and grain response arcs appeared successively (shown in Fig. 4(d)) due to the reduced of 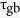 and 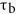. 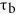 is so low that the bulk response does not work in the frequency range from 20 to 10^6^ Hz when the temperature rose to 300 K. The result is that the dielectric constant is maintained at a high level as large as 10^4^ and only the grain boundary response arc appeared in impedance spectroscopy in the applied frequency range (shown in Fig. 5(a)). In addition, 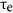 is small enough at room temperature, so the second plateau of dielectric permittivity appears at lower frequency. However, electrode arc can’t be separated from grain boundary arc in impedance spectroscopy because R_gb_ is much greater than R_e_^25^.

Fig. 5(a) and Fig. 5(c) show the impedance spectroscopy above 300 K and below 90 K, respectively. The bulk (R_b_) and grain boundary (R_gb_) resistances are thermally activated according to the equation:


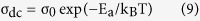


where σ_0_ is a constant, E_a_ is the activation energy, k_B_ is the Boltzmann constant, and T is the absolute temperature. The activation energies were calculated to be 0.052 eV and 0.35 eV for grain and grain boundary, as shown in Fig. 5(b) and (d). The grain activation energy could be attributed to the donor impurity energy level induced by In and Nb. The Nb doping into TiO_2_ will induce a shallow donor impurity energy level at 0.02-0.03 eV, accompanying the reduction of Ti^4+^^30^. However, because of the lack of oxygen vacancy at grain boundary, the electron emitted by doped-Nb ion will be captured by doped-In ion instead of Ti ion, leading to a sharp increase of resistance at grain boundary. Thus, the (In + Nb) co-doped TiO_2_ ceramic consists of semiconductor grains and insulating grain boundaries, and the colossal dielectric permittivity can be attributed to the microstructure. In other words, the IBLC mode works well in (In + Nb) co-doped TiO_2_ polycrystalline ceramic, instead of the electron-pinned defect-dipoles mode.

## Conclusions

In summary, 10% mol (In + Nb) co-doped TiO_2_ single crystal and polycrystalline ceramics were fabricated by the optical floating zone method and solid phase reaction method, respectively. The microstructure, valence state, dielectric response, and impedance spectroscopy of the samples were systematically investigated. Two dielectric relaxation processes were observed in the polycrystalline ceramics sample, while only one appeared in the single crystal sample. The high dielectric permittivity that appears at low frequency originated from the SBLC, because the dielectric behavior and impedance spectroscopy of single crystal sample depended on the testing voltage. The microstructure composed of semiconductor grains and insulating grain boundaries in the polycrystalline ceramics. The grain and grain boundary activation energies in the ceramic samples were calculated to be 0.052 eV and 0.35 eV from the impedance spectroscopy at different temperatures. Additionally, the relationship between the dielectric behavior and the electric response for the grain and grain boundary impedance spectroscopy indicates that the colossal dielectric permittivity in (In + Nb) co-doped TiO_2_ polycrystalline ceramics should be benefited from the IBLC instead of the electron-pinned defect-dipoles.

## Methods

The 10 mol % (In + Nb) co-doped TiO_2_ samples was synthesized using the standard conventional solid-state reaction (SCSS) method, where the rutile TiO_2_ (99.9%), Nb_2_O_5_ (99.99%) and In_2_O_3_ (99.99%) powders were used as raw materials. Powders were mixed and then uniaxially pressed to a disk. At last, the disk was sintered at 1500 °C in air for 20 h with a 2 °C/min. heating rate by the SCSS method. While the single crystal with the same composition was prepared by the optical floating zone method in an image furnace. Starting materials of dried TiO_2_, In_2_O_3_, and Nb_2_O_5_ were mixed into a test tube, typically 6 mm in diameter and 100 mm long, and heated at 1500 °C for 20 hours after being hydraulic pressed under an isostatic pressure of 70 MPa. The single crystal was grown in the air by the rate of 10 mm/h.

The phase of samples was characterized by X-ray diffraction (XRD, X’PERT PRO MPD, Holland).X-ray photoelectron spectroscopy (XPS, ESCALAB 250Xi, USA) was used to analyze the valence state of different elements. The microstructure was investigated by scanning electron microscopy (SEM, FEI PHENOM G1). The dielectric properties and the impedance spectroscopy were measured using Agilent 4980 A in PPMS (Physical Property Measurement System) and stove at low and high temperature, respectively.

## Additional Information

**How to cite this article**: Song, Y. *et al.* Origin of colossal dielectric permittivity of rutile Ti_0.9_In_0.05_Nb_0.05_O_2_: single crystal and polycrystalline. *Sci. Rep.* **6**, 21478; doi: 10.1038/srep21478 (2016).

## Supplementary Material

Supplementary Information

## References

[b1] Krohns S. *et al.* Theroute to resource-efficient novel materials. Nat. Mater. 10, 899–901 (2011). 22109596 10.1038/nmat3180

[b2] Buscaglia M. T. *et al.* High dielectric constant and frozen macroscopic polarization in dense nanocrystalline BaTiO_3_ ceramics. Phys. Rev. B. 73, 064114 (2006).

[b3] Homes C. C., Vogt T., Shapiro S. M., Wakimoto S. & Ramirez A. P. Optical response of high- dielectric-constant perovskite-related oxide. Science. 293, 673–676 (2001). 11474105 10.1126/science.1061655

[b4] Sinclair D. C., Adams T. B., Morrison F. D. & West A. R. CaCu_3_Ti_4_ O_12_: One-step internal barrier layer capacitor. Appl. Phys. Lett. 80, 2153–2155 (2002).

[b5] Wu J. B., Nan C. W. & Lin Y. H. & Yuan Deng. Giant Dielectric Permittivity Observed in Li and Ti Doped NiO. Phys. Rev. Lett. 89, 217601 (2002). 12443449 10.1103/PhysRevLett.89.217601

[b6] Liu X., Fan H. Q., Shi J. & Li Q. Origin of anomalous giant dielectric performance in novel perovskite: Bi_0.5−*x*_La_*x*_Na_0.5−*x*_Li_*x*_Ti_1−*y*_*M*_*y*_O_3_ (*M*=Mg^2+^, Ga^3+^). Sci. Rep 5, 12699 (2015). 26239525 10.1038/srep12699 PMC4523863

[b7] Zheng H., Weng W. J., Han G. R. & Du P. V. Colossal Permittivity and Variable-Range- Hopping Conduction of Polarons in Ni_0.5_Zn_0.5_Fe_2_O_4_ Ceramic. J. Phys. Chem. C 117, 12966–12972 (2013).

[b8] Li X. L. *et al.* High pressure treated ZnO ceramics towards giant dielectric constants. J. Mater. Chem. A 2, 16740–16745 (2014).

[b9] Sagdeo A. *et al.* Large dielectric permittivity and possible correlation between magnetic and dielectric properties in bulk BaFeO_3−δ_. Appl. Phys. Lett 105, 042906 (2014).

[b10] Zheng T. *et al.* Potassium–sodium niobate lead-free ceramics: modified strain as well as piezoelectricity. J. Mater. Chem. A 3, 1868–1874 (2015).

[b11] Guillemet-Fritsch S. *et al.* Colossal permittivity in ultrafine grain size BaTiO_3-x_ and Ba_0.95_La_0.05_TiO_3-x_ materials. Adv. Mater. 20, 551–555 (2008).

[b12] Hu W. B. *et al.* Electron-pinned defect-dipoles for high-performance colossal permittivity materials. Nat. Mater 12, 821–827 (2013). 23812129 10.1038/nmat3691

[b13] Cheng X. J., Li Z. W. & Wu J. G. Colossal permittivity in ceramics of TiO_2_ Co-doped with niobium and trivalent cation. J. Mater. Chem. A 3, 5805–5810 (2015).

[b14] Hu W. B. *et al.* Colossal Dielectric Permittivity in (Nb+Al) Codoped Rutile TiO_2_ Ceramics: Compositional Gradient and Local Structure. Chem. Mater. 27, 4934–4942 (2015).

[b15] Gai Z. G. *et al.* A colossal dielectric constant of an amorphous TiO_2_:(Nb, In) film with low loss fabrication at room temperature. J. Mater. Chem. C 2, 6790–6795 (2014).

[b16] Han H. *et al.* Quasi-intrinsic colossal permittivity in Nb and In co-doped rutile TiO_2_ nanoceramics synthesized through a oxalate chemical-solution route combined with spark plasma sintering. Phys. Chem. Chem. Phys. 17, 16864–16875 (2015). 26058428 10.1039/c5cp02653a

[b17] Zhao X. G. *et al.* Origin of colossal permittivity in (In_1/2_Nb_1/2_) TiO_2_ via broadband dielectric spectroscopy. Phys. Chem. Chem. Phys. 17, 23132—23139 (2015). 26278381 10.1039/c5cp02741a

[b18] Li J. L. *et al.* Microstructure and dielectric properties of (Nb+In) co-doped rutile TiO_2_ ceramics. J. Appl. Phys 116, 074105 (2014).

[b19] Li J. L. *et al.* Evidences of grain boundary capacitance effect on the colossal dielectric permittivity in (Nb+In) co-doped TiO_2_ ceramics. Sci. Rep, 5, 8295 (2015). 25656713 10.1038/srep08295 PMC4319151

[b20] Li J. L., Li F., Xu Z., Zhuang Y. Y. & Zhang S. J. Nonlinear I–V behavior in colossal permittivity ceramic: (Nb+In) co-doped rutile TiO_2_. Ceramics International 41. 798–803 (2015).

[b21] Wang X. J. *et al.* Origin of ferromagnetism in aluminum-doped TiO_2_ thin films: Theory and experiments. Appl. Phys. Lett 105, 262402 (2014).

[b22] Yang J. Y. *et al.* d carrier induced intrinsic room temperature ferromagnetism in Nb:TiO_2_ film. Appl. Phys. Lett 100, 202409 (2012).

[b23] Erdem B. *et al.* XPS and FTIR Surface Characterization of TiO_2_ Particles Used in Polymer Encapsulation. Langmuir 17, 2664–2669 (2001).

[b24] Ramos-Moore E., Ferrari P., Diaz-Droguett D. E., Lederman D. & Evans J. T. Raman and x-ray photoelectron spectroscopy study of ferroelectric switching in Pb(Nb,Zr,Ti)O_3_ thin films. J. Appl. Phys 111, 014108 (2012).

[b25] Li M. *et al.* Origin(s) of the apparent high permittivity in CaCu_3_Ti_4_O_12_ ceramics: clarification on the contributions from internal barrier layer capacitor and sample-electrode contact effects. J. Appl. Phys 106, 104106 (2009).

[b26] Krohns S., Lunkenheimer P., Ebbinghaus S. G. & Loidl A. Broadband dielectric spectroscopy on single-crystalline and ceramic CaCu_3_Ti_4_O_12_. Appl. Phys. Lett 91, 022910 (2007).

[b27] Krohns S., Lunkenheimer P., Ebbinghaus S. G. & Loidl A. Colossal dielectric constants in single-crystalline and ceramic CaCu_3_Ti_4_O_12_ investigated by broadband dielectric spectroscopy. J. Appl. Phys 103, 084107 (2008).

[b28] Shen M. R., Ge S. B. & Cao W. W. Dielectric enhancement and Maxwell-Wagner effects in polycrystalline ferroelectric multilayered thin films. J. Phys. D: Appl. Phys 34, 2935–2938 (2001).

[b29] Ren S. Q. *et al.* Coexistence of electric field controlled ferromagnetism and resistive switching for TiO_2_ film at room temperature. Appl. Phys. Lett 107, 062404 (2015).

[b30] Morris D., Dou Y., Rebane J., Mitchell C. E. J. & Egdell R. G. Photoemission and STM study of the electronic structure of Nb-doped TiO_2_. Phys. Rev. B 61, 13445–13457 (2000).

