# Supplementary material for: Origin of colossal dielectric permittivity of rutile Ti0.9In0.05Nb0.05O2: single crystal and polycrystalline
Source: Sci Rep. 2016 Feb 12;6:21478. doi: 10.1038/srep21478 (PMC4751469; doi:10.1038/srep21478)
Supplement: Supplementary Information [file srep21478-s1.doc]

**Supporting Information**

**Origin of colossal dielectric permittivity of rutile Ti_0.9_In_0.05_Nb_0.05_O_2_**: **single crystal and polycrystalline**

Yongli Song^1^, Xianjie Wang1[[1]](#footnote-2)a), Yu Sui1,[[2]](#footnote-3)a), Ziyi Liu^1^, Yu Zhang^1^, Hongsheng Zhan^1^, Bingqian Song^1^, Zhiguo Liu^1^, Zhe Lv^1^, Lei Tao^1^ and Jinke Tang^2^

^1^ Department of Physics, Harbin Institute of Technology, Harbin 150001, People’s Republic of China.

^2^Department of Physics & Astronomy, University of Wyoming, Laramie, WY 82071, USA

**Table S1. ICP analysis of single crystal and ceramics**

|  | In (mg/L) | Nb(mg/L) | Ti(mg/L) |
| --- | --- | --- | --- |
| Single crystal | 0.035 | 0.028 | 0.263 |
| Ceramics | 0.032 | 0.026 | 0.239 |

The single crystal and ceramics sample were ground into powder, 5mg power was dissolved in 1L hydrofluoric which is diluted by 100ml 30% hydrofluoric, respectively. After heating for one night at 80℃, solution is diluted ten times again to take the ICP test (PerkinElmer, ICP-OES 5300DV). The analytical wavelength for In, Nb and Ti are 230.606nm, 313.079 and 334.940nm, respectively. In accordance with the calculation, the mole ratio of In, Nb and Ti in both single crystal and ceramics is 1:1:18 with the consistent design. The little different on the quality score between single crystal and ceramics may originate from the weighing of the powder and the dilution of the solution.

**Fig S1. Valence states of the elements Ti (a) and O(b) of the TiO_2_ ceramics co-doped with 10% (In+Nb).** The XPS results of Ti 2p in the ceramics reveals the existence of Ti^3+^ ions, giving a Ti^3+^/Ti proportion of ~5.2%, which is very similar to the single crystal results. The data of O also very similar to that of single crystal.

1. ^a^Author to whom correspondence should be addressed; Email: [wangxianjie@hit.edu.cn](mailto:wangxianjie@hit.edu.cn) (X. Wang) ; [suiyu@hit.edu.cn](mailto:suiyu@hit.edu.cn) (Y.Sui) [↑](#footnote-ref-2)
2. ^a^ [↑](#footnote-ref-3)
